# Supplementary figures and images for: Phage-like particle vaccines are highly immunogenic and protect against pathogenic coronavirus infection and disease
Source: NPJ Vaccines. 2022 May 26;7:57. doi: 10.1038/s41541-022-00481-1 (PMC9135756; doi:10.1038/s41541-022-00481-1)

**Fig 2A**

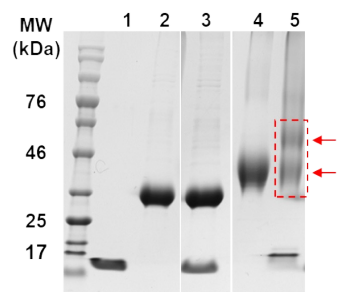

**Fig 2B**

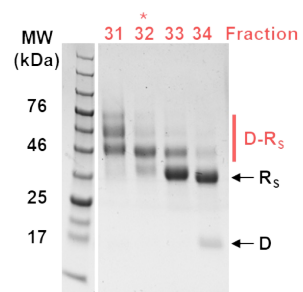

**Fig 2C**

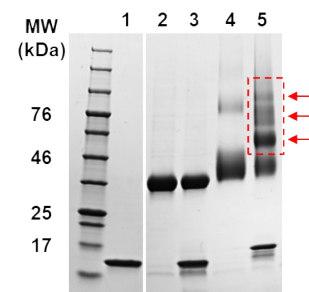

**Fig 2D**

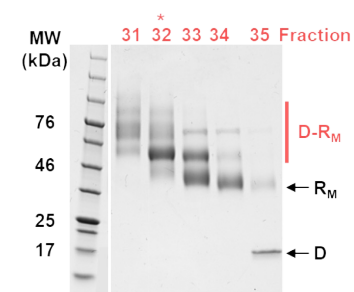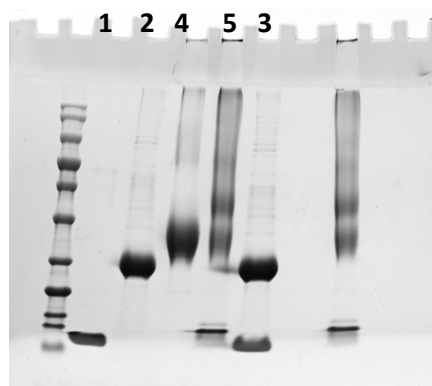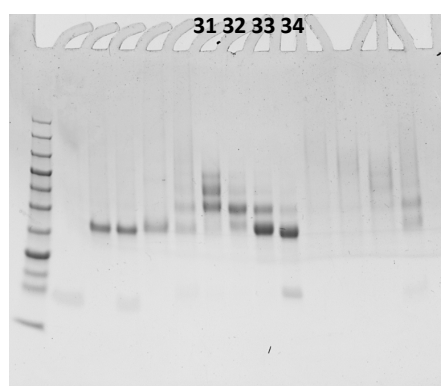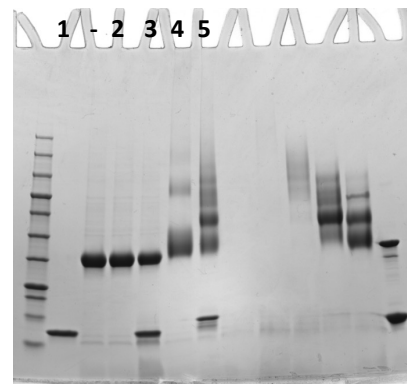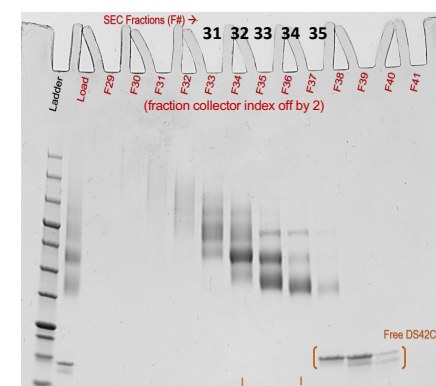

**Fig 3A**

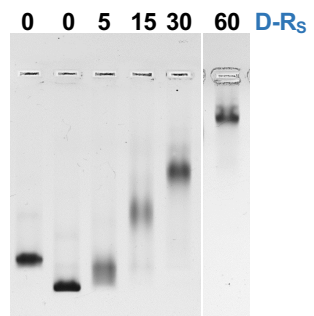

**Fig 3B**

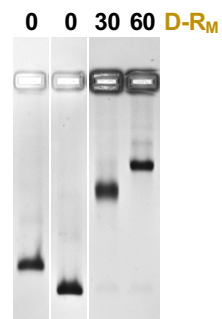

**Fig 3C**

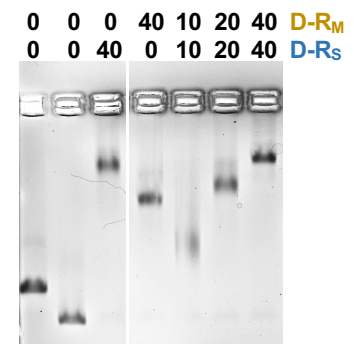

0 0 5 15 30

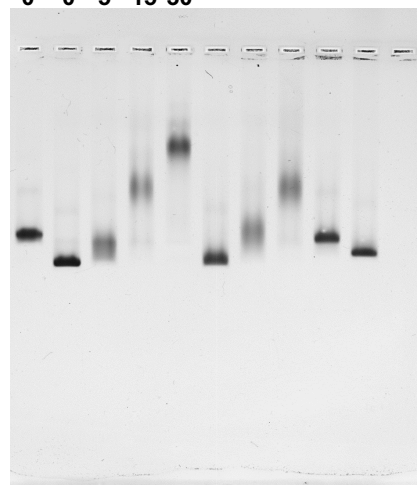

60

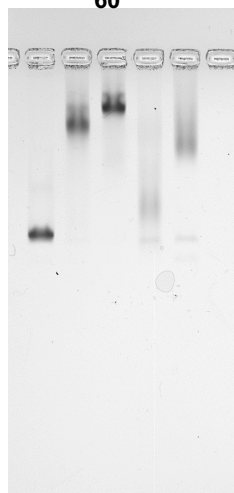

0 0 30 60

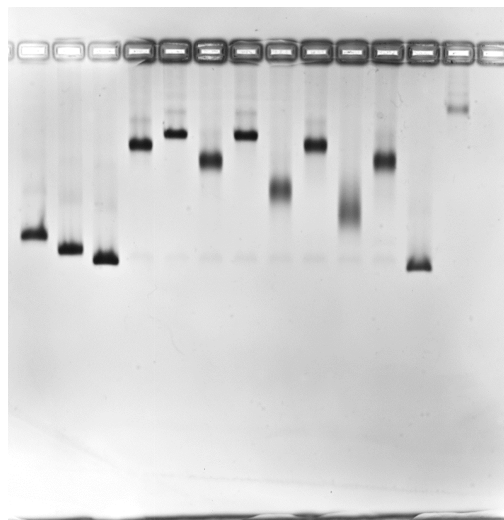

0 0 0 40 10 20 40  $D-R_M$   
0 0 40 0 10 20 40  $D-R_S$

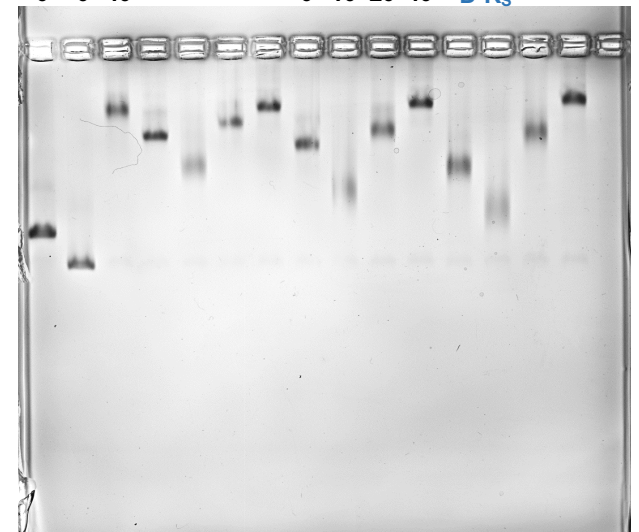

Supplement: Supplementary file 1 — Dataset 1 [file 41541_2022_481_MOESM1_ESM.pdf]
