# Supplementary material for: Phage-like particle vaccines are highly immunogenic and protect against pathogenic coronavirus infection and disease
Source: NPJ Vaccines. 2022 May 26;7:57. doi: 10.1038/s41541-022-00481-1 (PMC9135756; doi:10.1038/s41541-022-00481-1)
Supplement: Supplementary file 2 — Supplemental Material [file 41541_2022_481_MOESM2_ESM.pdf]

Supplementary Table 1: SARS-CoV-2 qPCR primer and probe combinations

SARS-CoV-2 E gene qPCR primer and probe

E Sarbeco fwd 1	5'	ACAGGTACGTTAATAGTTAATAGCGT	3'
E Sarbeco rev 2	5'	ATATTGCAGCAGTACGCACACA	3'
E Sarbeco probe 1	5'	FAM-ACACTAGCCATCCTTACTGCGCTTCG-BHQ	3'

SARS-CoV-2 N (sgRNA) qPCR primer and probe

sgN SARS2 fwd	5'	ACCTTCCCAGGTAACAAACCAACCA	3'
sgN SARS2 rev	5'	CACCAAACGTAATGCGGGGTGC	3'
sgN SARS2 probe	5'	FAM-CTGATAATGGACCCCAAAATCAGCGA-BHQ	3'

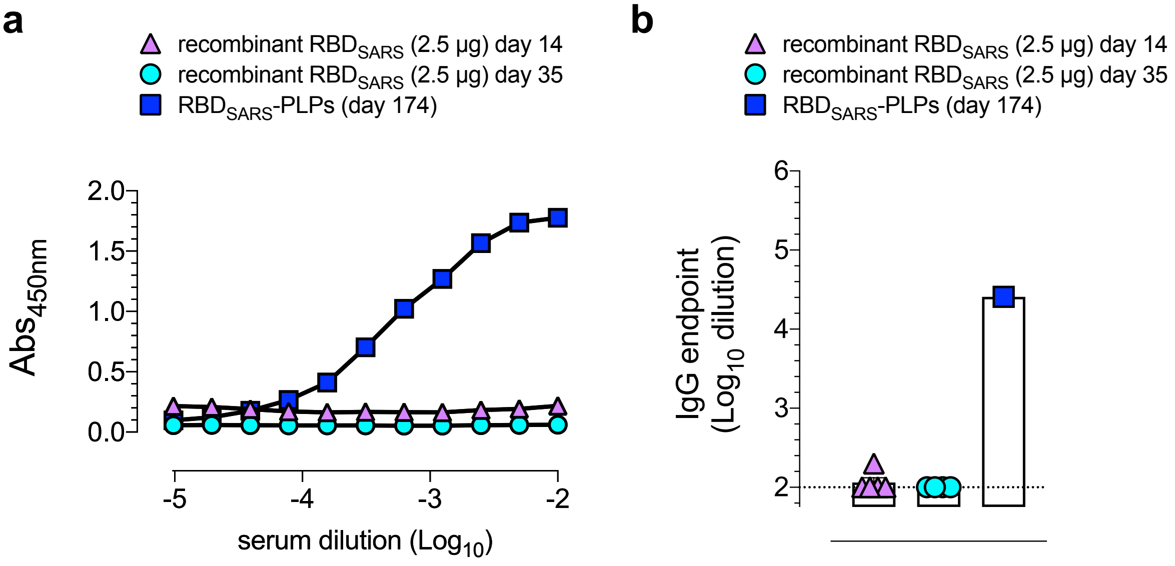

Supplementary Figure 1. Recombinant RBD_{SARS} alone is insufficient to induce RBD-specific IgG responses in mice. WT BALB/c mice (n = 4-5) were immunized with 2.5 µg of recombinant RBD_{SARS} by i.m. injection on days 0 and 21. Serum was collected at day 14 and day 35 (14 days post boost) and the presence of serum RBD_{SARS}-specific IgG was determined by ELISA. (**A**) Absorbance at 450 nm of serum samples was determined by ELISA. The assay was confirmed by inclusion of a day 174 serum sample from BALB/c mice immunized i.m. with 10 µg of RBD_{SARS}-PLPs from the experiment described in Figure 4B. Each point represents the mean of 5 mice, and error bars represent the mean ± SEM. (**B**) Serum IgG endpoint titers from mice immunized with 2.5 µg of recombinant RBD_{SARS} and control serum were determined by ELISA. Each point represents an individual mouse, and error bars represent the mean ± SEM.

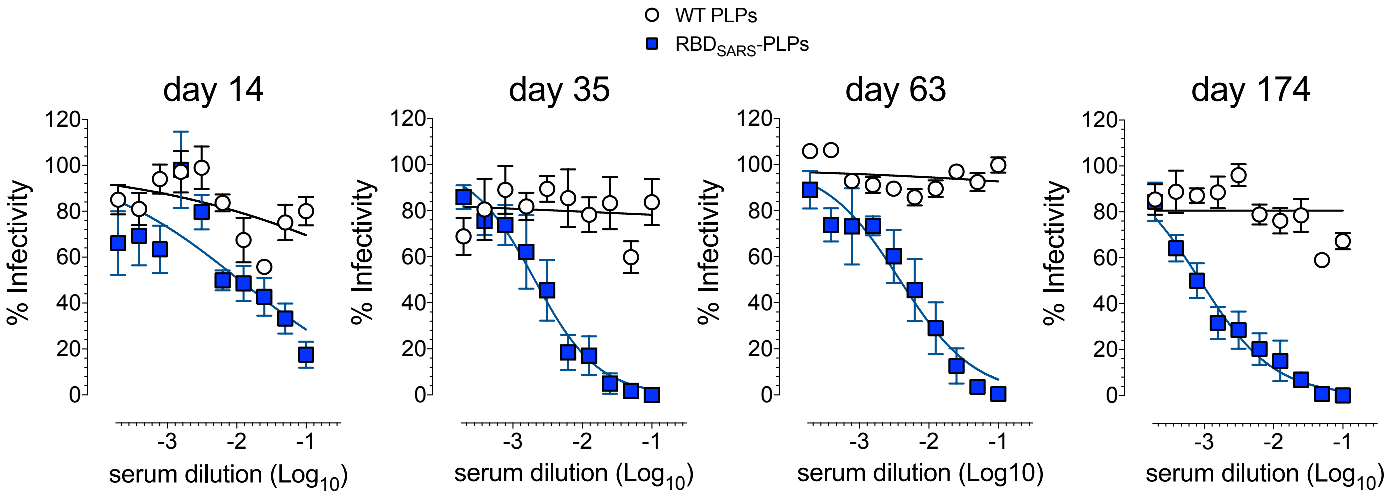

Supplementary Figure 2. Immunization with RBD_{SARS}-PLPs elicits potent and durable SARS-CoV-2 neutralizing antibodies. WT BALB/c mice (n = 5/group) were immunized with 10 µg of WT PLPs (control) or 60% RBD_{SARS}-PLPs by intramuscular (i.m.) injection on days 0 and 21. Animals were bled on days 14, 35, 63, and 174 and SARS-CoV-2 neutralizing activity was determined by a focus reduction neutralization test (FRNT). Each point represents the mean of 5 mice, and error bars represent the mean ± SEM.

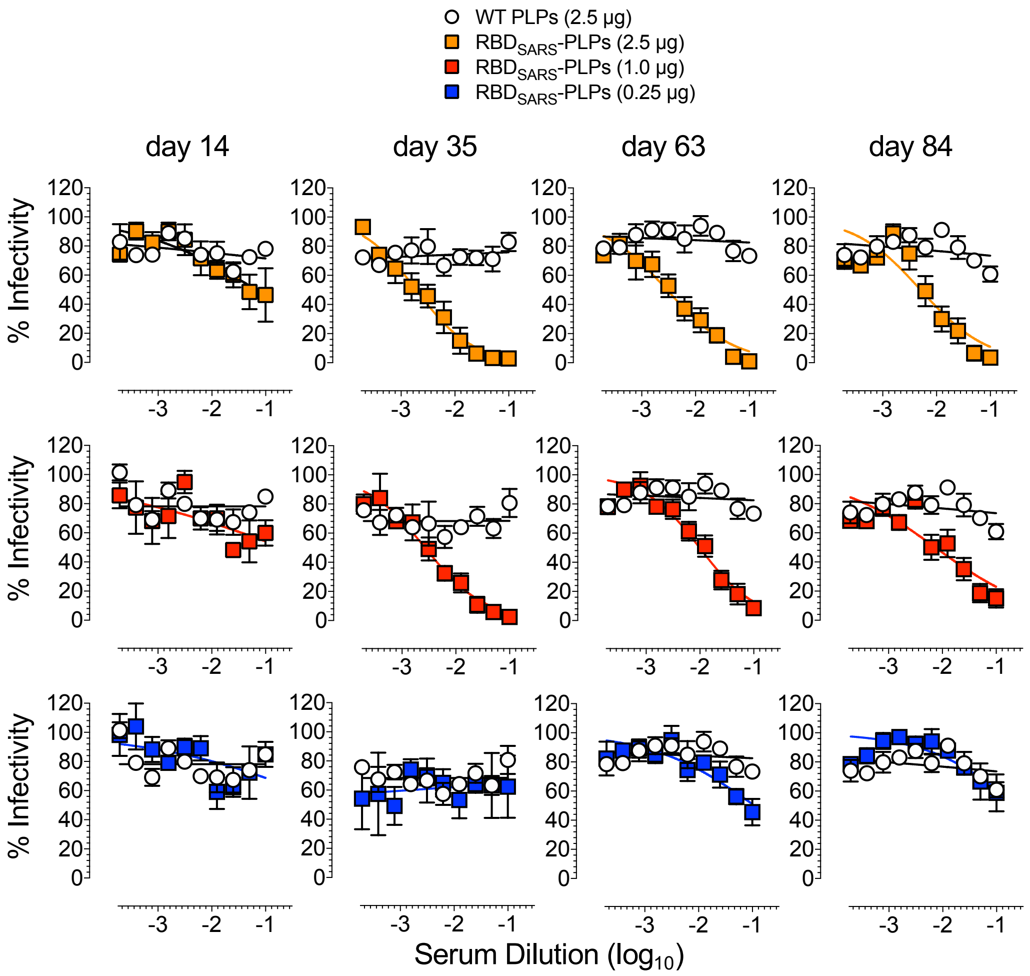

Supplementary Figure 3. Low dose immunization with RBD$_{SARS}$-PLPs elicits potent and durable SARS-CoV-2 antibody responses. WT BALB/c mice (n = 5/group) were immunized with 2.5 µg of WT PLPs (control) or 2.5, 1.0, or 0.25 µg of RBD$_{SARS}$-PLPs by intramuscular (i.m.) injection on days 0 and 21. Animals were bled on days 14, 35, 63, and 84. SARS-CoV-2 neutralizing activity in serum samples was determined by a focus reduction neutralization test (FRNT). Each point represents the mean of 5 mice, and error bars represent the mean ± SEM.
