# Supplementary material for: Phage-like particle vaccines are highly immunogenic and protect against pathogenic coronavirus infection and disease
Source: NPJ Vaccines. 2022 May 26;7:57. doi: 10.1038/s41541-022-00481-1 (PMC9135756; doi:10.1038/s41541-022-00481-1)
Supplement: Supplementary file 3 — REPORTING SUMMARY [file 41541_2022_481_MOESM3_ESM.pdf]

nature portfolio

Corresponding author(s): Thomas Morrison

Last updated by author(s): Apr 7, 2022

Reporting Summary

Nature Portfolio wishes to improve the reproducibility of the work that we publish. This form provides structure for consistency and transparency in reporting. For further information on Nature Portfolio policies, see our Editorial Policies and the Editorial Policy Checklist.

Statistics

For all statistical analyses, confirm that the following items are present in the figure legend, table legend, main text, or Methods section.

n/a	Confirmed	
☐	☒	The exact sample size (n) for each experimental group/condition, given as a discrete number and unit of measurement
☐	☒	A statement on whether measurements were taken from distinct samples or whether the same sample was measured repeatedly
☐	☒	The statistical test(s) used AND whether they are one- or two-sided<br>*Only common tests should be described solely by name; describe more complex techniques in the Methods section.*
☒	☐	A description of all covariates tested
☐	☒	A description of any assumptions or corrections, such as tests of normality and adjustment for multiple comparisons
☐	☒	A full description of the statistical parameters including central tendency (e.g. means) or other basic estimates (e.g. regression coefficient) AND variation (e.g. standard deviation) or associated estimates of uncertainty (e.g. confidence intervals)
☒	☐	For null hypothesis testing, the test statistic (e.g. F, t, r) with confidence intervals, effect sizes, degrees of freedom and P value noted<br>*Give P values as exact values whenever suitable.*
☒	☐	For Bayesian analysis, information on the choice of priors and Markov chain Monte Carlo settings
☒	☐	For hierarchical and complex designs, identification of the appropriate level for tests and full reporting of outcomes
☒	☐	Estimates of effect sizes (e.g. Cohen's d, Pearson's r), indicating how they were calculated

Our web collection on statistics for biologists contains articles on many of the points above.

Software and code

Policy information about availability of computer code

Data collection	EM images were processed in Fiji
Data analysis	Graphpad Prism version 9.1.2. was used for statistical analysis and graphical representation.

For manuscripts utilizing custom algorithms or software that are central to the research but not yet described in published literature, software must be made available to editors and reviewers. We strongly encourage code deposition in a community repository (e.g. GitHub). See the Nature Portfolio guidelines for submitting code & software for further information.

Data

Policy information about availability of data

All manuscripts must include a data availability statement. This statement should provide the following information, where applicable:

- Accession codes, unique identifiers, or web links for publicly available datasets
- A description of any restrictions on data availability
- For clinical datasets or third party data, please ensure that the statement adheres to our policy

All data are available in the article and supplementary information. Source data of all data presented in graphs within the figures will be provided with the paper.

Field-specific reporting

Please select the one below that is the best fit for your research. If you are not sure, read the appropriate sections before making your selection.

☒ Life sciences ☐ Behavioural & social sciences ☐ Ecological, evolutionary & environmental sciences

For a reference copy of the document with all sections, see nature.com/documents/nr-reporting-summary-flat.pdf

Life sciences study design

All studies must disclose on these points even when the disclosure is negative.

Sample size	Sample size calculations were not performed. Samples sizes were determined by effect sizes and intragroup variability n pilot studies that demonstrated statistical significance had been achieved with 5 mice per group.
Data exclusions	No data were excluded.
Replication	RBD-SARS immunizations were replicated multiple times throughout the study. RBD-MERS immunization experiments were replicated across monovalent and bivalent particles.
Randomization	Mice were not randomized into experimental groups.
Blinding	Investigators performing immunization and antibody response analysis were not blinded. Investigators performing virulent virus challenge were blinded to the experimental groups.

Reporting for specific materials, systems and methods

We require information from authors about some types of materials, experimental systems and methods used in many studies. Here, indicate whether each material, system or method listed is relevant to your study. If you are not sure if a list item applies to your research, read the appropriate section before selecting a response.

Materials & experimental systems

n/a	Involved in the study	
☐	☒	Antibodies
☐	☒	Eukaryotic cell lines
☒	☐	Palaeontology and archaeology
☐	☒	Animals and other organisms
☒	☐	Human research participants
☒	☐	Clinical data
☒	☐	Dual use research of concern

Methods

n/a	Involved in the study	
☒	☐	ChIP-seq
☒	☐	Flow cytometry
☒	☐	MRI-based neuroimaging

Antibodies

Antibodies used	1. chimeric human antiSARS-CoV spike antibody clone CR3022 (Absolute Antibody, Ab01680)<br>2. mouse anti-MERS-CoV spike antibody clone D12 (Absolute Antibody, Ab00696)<br>3. goat anti-mouse IgG-HRP (Southern Biotech, 1030-05)<br>4. goat anti-human IgG Fc-HRP (Southern Biotech, 2014-05)<br>5. goat anti-mouse IgG1-BIOT (Southern Biotech, 1071-08)<br>6. goat anti-mouse IgG2a Human ads-BIOT (Southern Biotech, 1080-08)<br>7. goat anti-mouse IgG2b-BIOT (Southern Biotech, 1091-08)<br>8. goat anti-mouse IgG3 human ads-BIOT (Southern Biotech, 1100-08)<br>9. streptavidin-HRP (Southern Biotech, 7100-05)
Validation	1. Chimeric human antiSARS-CoV spike antibody clone CR3022 (Absolute Antibody, Ab01680) validation statement from manufacturer "This antibody binds to both SARS-CoV and SARS-CoV-2 with high affinity (PMID: 16796401 & 32065055)". This antibody was also validated for cross-reactivity against SARS-CoV-2 RBD generated in our lab by ELISA (see figure 3F).<br><br>2. Mouse anti-MERS-CoV spike antibody clone D12 (Absolute Antibody, Ab00696) validation statement from manufacturer "This antibody binds to MERS-CoV spike protein receptor binding domain on the S1 protein. Wang et al. Evaluation of candidate vaccine approaches for MERS-CoV Nat Commun 6, 7712 (2015) PMID:26218507. This antibody was also validated for cross-reactivity against MERS-CoV RBD generated in our lab by ELISA (see figure 3G).

Eukaryotic cell lines

Policy information about cell lines

Cell line source(s)	Vero E6 (CRL-1586, American Type Culture Collection (ATCC))
Authentication	Cells were authenticated by ATCC
Mycoplasma contamination	All cells tested negative for mycoplasma contamination
Commonly misidentified lines (See ICLAC register)	No commonly misidentified cell lines were used

Animals and other organisms

Policy information about studies involving animals; ARRIVE guidelines recommended for reporting animal research

Laboratory animals	Mouse, strain Balb/c (JAX 000651), females aged 6-8 weeks
Wild animals	None used.
Field-collected samples	None used.
Ethics oversight	Animal studies were carried out in accordance with the recommendations in the Guide for the Care and Use of Laboratory Animals of the National Institutes of Health. Animal use protocols were approved by the Institutional Animal Care and Use Committees at the University of Colorado School of Medicine (Assurance number A3269-01) and the University of Maryland School of Medicine (Assurance number D16-00125(A3200-01)).

Note that full information on the approval of the study protocol must also be provided in the manuscript.
